# Glycocalyx degradation and the endotheliopathy of viral infection

**DOI:** 10.1371/journal.pone.0276232

**Published:** 2022-10-19

**Authors:** Sharven Taghavi, Sarah Abdullah, Farhana Shaheen, Lauren Mueller, Brennan Gagen, Juan Duchesne, Chad Steele, Derek Pociask, Jay Kolls, Olan Jackson-Weaver

**Affiliations:** 1 Department of Surgery, Tulane University School of Medicine, New Orleans, Louisiana, United States of American; 2 Department of Microbiology, Tulane University School of Medicine, New Orleans, Louisiana, United States of American; 3 Department of Internal Medicine, Tulane University School of Medicine, New Orleans, Louisiana, United States of American; 4 Center for Translational Research in Infection and Inflammation, Tulane University School of Medicine, New Orleans, Louisiana, United States of American; Eötvös Loránd Research Network Biological Research Centre, HUNGARY

## Abstract

The endothelial glycocalyx (EGX) contributes to the permeability barrier of vessels and regulates the coagulation cascade. EGX damage, which occurs in numerous disease states, including sepsis and trauma, results in endotheliopathy. While influenza and other viral infections are known to cause endothelial dysfunction, their effect on the EGX has not been described. We hypothesized that the H1N1 influenza virus would cause EGX degradation. Human umbilical vein endothelial cells (HUVECs) were exposed to varying multiplicities of infection (MOI) of the H1N1 strain of influenza virus for 24 hours. A dose-dependent effect was examined by using an MOI of 5 (n = 541), 15 (n = 714), 30 (n = 596), and 60 (n = 653) and compared to a control (n = 607). Cells were fixed and stained with FITC-labelled wheat germ agglutinin to quantify EGX. There was no difference in EGX intensity after exposure to H1N1 at an MOI of 5 compared to control (6.20 vs. 6.56 Arbitrary Units (AU), p = 0.50). EGX intensity was decreased at an MOI of 15 compared to control (5.36 vs. 6.56 AU, p<0.001). The degree of EGX degradation was worse at higher doses of the H1N1 virus; however, the decrease in EGX intensity was maximized at an MOI of 30. Injury at MOI of 60 was not worse than MOI of 30. (4.17 vs. 4.47 AU, p = 0.13). The H1N1 virus induces endothelial dysfunction by causing EGX degradation in a dose-dependent fashion. Further studies are needed to characterize the role of this EGX damage in causing clinically significant lung injury during acute viral infection.

## Introduction

The endothelial glycocalyx is a glycoprotein layer on the luminal side of vascular endothelial cells [1]. Healthy endothelial glycocalyx maintains vessel permeability and regulates the coagulation cascade in response to inflammation, mechanical disturbances, and vascular pathophysiology [2]. Damage to the endothelial glycocalyx contributes to pathological clinical presentations, such as edema due to increased vascular permeability, hypercoagulability due to increased platelet aggregation, and endotheliopathy due to inflammation [2,3]. Endothelial glycocalyx damage has been implicated in many disease processes, such as acute kidney injury, hemorrhagic shock, diabetes, and sepsis [4–7]. The COVID-19 pandemic has led to increased interest in the effects of viral infection on the endothelial glycocalyx layer. COVID-19 acts through ACE2 receptors on vascular endothelial cells, leading to glycocalyx damage [8–11]. Studies have shown that glycocalyx degradation plays a key role in the development of viral pneumonia or acute respiratory distress syndrome (ARDS) [12–14].

Influenza A is a major cause of morbidity and mortality worldwide [15,16]. The effects of H1N1 viral infection on the endothelial glycocalyx are not fully understood. Multiple studies have demonstrated that endothelial glycocalyx degradation products are present in a more severe disease state after viral infection. Benatti et al. showed that hyaluronan levels above 19 ng/ml in patients with flu syndrome were associated with a significant increase in the 28-day mortality rate [17]. Huang et al. found that increased plasma syndecan-1 levels was an independent risk factor for mortality in patients with H1N1 [18]. Injury to endothelial cells triggers endothelial glycocalyx degradation and shedding, resulting in an increase in vascular permeability and pulmonary capillary leak [19,20].

Despite the knowledge of degradation products being present in more severe disease states, the mechanism by which H1N1 damages the glycocalyx is poorly defined. In this study, we set out to determine the effect of H1N1 influenza virus on endothelial glycocalyx using human umbilical vein endothelial cells (HUVECs). We hypothesized that H1N1 infection would result in endothelial glycocalyx shedding.

## Methods

### HUVEC culture

Human umbilical vein endothelial cells were obtained from the American Type Culture Collection. HUVECs were grown in M200 medium supplemented with low serum growth supplement and penicillin/streptomycin on 2% gelatin-coated 10-cm plastic dishes in a cell culture incubator at 37°C with 5% CO_2_ atmosphere as previously described [3,21]. Cells were subsequently passaged by digestion in 0.25% trypsin solution after attainment of approximately 80% confluence. Cells used for experiments were done so between passages 1 and 3. For endothelial glycocalyx staining intensity quantification, using established techniques, [21] HUVECs were plated in gelatin-coated 96-well plastic cell culture plates, at a confluence of approximately 90%. 1% bovine serum albumin (BSA) was added to the M200–low serum growth supplement–penicillin/streptomycin to support glycocalyx growth [3,21,22]. Cells were subsequently cultured for 24 hours to allow glycocalyx development prior to virus exposure.

### Experimental design

To investigate the effects of H1N1 influenza on the EG, cultured HUVECs were exposed to A/PR/8/34 H1N1 (PR8) from a frozen stock. H1N1 virus was administered to the HUVECs at 0, 5, 15, 30, or 60 MOI (multiplicity of infection) directly to culture media (n = 607, 541, 714, 596, or 653 cells, respectively) for a total of 24 hours. Marimastat (R&D Systems) was administered to cells at final concentration of 1 μM (n = 122). Oseltamivir (R&D Systems) was administered at 10 μM (n = 120). Recombinant H1N1 neuraminidase (R&D Systems) was administered at a final concentration of 0.25 μg/100 ml (n = 1369). Sample sizes were determined based on previous studies demonstrating degree of glycocalyx degradation after exposing HUVECs to injury [3,21].

### Glycocalyx quantification

Glycocalyx staining of HUVECs was then performed as previously described [3,21,23]. After completion of the H1N1 or neuraminidase exposure, cells concentrated formaldehyde solution was applied directly to the culture medium for fixation to yield a final formaldehyde concentration of 3.5%. After fixation for 10 minutes, cells were then washed with phosphate-buffered saline (PBS) supplemented with 1% BSA. Cells were then stained with 23 μg/mL wheat germ agglutinin (WGA) and 23 μg/mL 4′,6-diamidino-2-phenylindole (DAPI) in PBS with 1% BSA for 20 minutes at room temperature in the dark. Staining was performed for this short time period to ensure that there is no penetration of the wheat germ agglutin into the cytoplasm, confounding results with non-surface layer staining. Cells were then washed twice with 1% BSA in PBS and covered with Fluoro-Gel mounting medium (Electron Microscopy Sciences, Hatfield, PA). Glycocalyx and nuclei (DAPI) were imaged on an EVOS fluorescence microscope (Thermofisher, Waltham, MA) under identical conditions. Three to ten images were taken of each condition, with approximately 150–200 cells per image. ImageJ software (National Institutes of Health, Bethesda, MD) was used to quantify glycocalyx fluorescence intensity overlaying the nuclei of each visible cell. Phase contrast images were also taken to assess overt cytopathology and apoptosis and to determine HUVEC viability.

### SDS-PAGE western blots for MMP9

HUVECs were lysed in lysis buffer (50 mM Tris-HCl, pH 7.5, 150 mM NaCl, 0.5 M ethylenediamine tetraacetic acid [EDTA], 1% Triton X-100, and Halt protease inhibitor cocktail [Thermofisher, Waltham, MA]). Proteins were then quantified using Bio-Rad protein assay (Bio-Rad Laboratories, Hercules, CA), and 20 μg of protein was separated by sodium dodecyl sulfate (SDS)–polyacrylamide gel electrophoresis (SDS-PAGE) on a 4% to 12% gradient acrylamide gel run at 100 V. Proteins were then transferred to 0.45-μm polyvinylidene difluoride (PVDF) membrane at 30 V for 2 hours. Membranes were blocked in Tris-buffered saline (137 mM NaCl, 20 mM Tris base), 0.1% Tween 20, and 5% BSA (blocking solution) for 1 hour, followed by overnight incubation with primary antibody diluted 1:1000 in Tris-buffered saline, 0.1% Tween 20, and 3% BSA, and 1-hour incubation with horseradish peroxidase-conjugated secondary antibody diluted at 1:5,000. The primary antibody used for matrix metalloproteinase 9 (MMP9) was rabbit monoclonal antibody D6O3H (Cell Signaling Technology, Danvers, MA). Immunoreactive protein was detected using ECL (GE Healthcare, Boston, MA) imaged on a Bio-Rad ChemiDoc MP Imaging System (Bio-Rad, Hercules, CA). Bands size was quantified using ImageJ (National Institutes of Health, Bethesda, MD).

### Gelatin zymography

HUVECs were lysed in lysis buffer (50 mM Tris-HCl, pH 7.5, 150 mM NaCl, 0.5 M ethylenediamine tetraacetic acid [EDTA], 1% Triton X-100, and Halt protease inhibitor cocktail [Thermofisher, Waltham, MA]). Proteins were quantified using Bio-Rad protein assay (Bio-Rad Laboratories, Hercules, CA), and 20 μg of protein was separated by non-reducing sodium dodecyl sulfate (SDS)–polyacrylamide gel electrophoresis (SDS-PAGE) on a 10% acrylamide gel containing 1 mg/ml gelatin run at 100 V. The gel was incubated with zymography incubation buffer (1% Triton X-100, 50 mM Tris-HCl, 5 mM CaCl_2_, 1 uM ZnCl_2_) overnight at 37°C. The gel was then stained with staining solution (40% methanol, 10% acetic acid, 0.5% coomassie blue in H_2_O) for 1 hour at room temperature, and destained (40% methanol, 10% acetic acid in H_2_O) until bands were clearly visualized. The gel was imaged on a Bio-Rad ChemiDoc MP Imaging System (Bio-Rad, Hercules, CA) and bands were quantified using ImageJ (National Institutes of Health, Bethesda, MD).

### Real-time quantitative reverse-transcription polymerase chain reaction

RNA was obtained with Trizol (Invitrogen) and used as a template for reverse transcriptase (iScript RT Supermix; Bio-Rad, Hercules, CA). Messenger RNAs (mRNAs) were quantified by real-time polymerase chain reaction with IQ SYBR Green Supermix (Bio-Rad, Hercules, CA) and normalized against *TUBB* mRNA as the internal control gene. Relative changes in expression were calculated using the ΔΔCt method as established in prior studies [24]. Primer sequences are listed in Table 1.

**Table 1:** Primer sequences used for PCR.

mRNA	Forward Primer	Reverse Primer
*PPIA*	5’-CCCACCGTGTTCTTCGACATT-3’	5’-GGACCCGTATGCTTTAGGATGA-3’
*ADAM15*	5’-CAGGACGATCTCCCAATTAGC-3’	5’-GGACCAACTCCCTATTCTGTAGC-3’
*ADAM17*	5’-GTGGATGGTAAAAACGAAAGCG-3’	5’-GGCTAGAACCCTAGAGTCAGG-3’
*MMP1*	5’-AAAATTACACGCCAGATTTGCC-3’	5’-GGTGTGACATTACTCCAGAGTTG-3’
*MMP2*	5’-TACAGGATCATTGGCTACACACC-3’	5’-GGTCACATCGCTCCAGACT-3’
*MMP7*	5’-GAGTGAGCTACAGTGGGAACA-3’	5’-CTATGACGCGGGAGTTTAACAT-3’
*MMP9*	5’-AGACCTGGGCAGATTCCAAAC-3’	5’-CGGCAAGTCTTCCGAGTAGT-3’
*MMP14*	5’-GGCTACAGCAATATGGCTACC-3’	5’-GATGGCCGCTGAGAGTGAC-3’
*MMP25*	5’-GACTGGCTGACTCGCTATGG-3’	5’-TGATGGCATCGCGCAACTT-3’
*SDC1*	5’-CTGCCGCAAATTGTGGCTAC-3’	5’-TGAGCCGGAGAAGTTGTCAGA-3’
*SDC2*	5’-TTGACAACAGCTCCATTGAAGAA-3’	5’-CAGCTCTGGACTCTCTACATCC-3’
*SDC3*	5’-TGGCGCAGTGAGAACTTCG-3’	5’-CCCCGAGTAGAGGTCATCCAG-3’
*SDC4*	5’-TCCCCACCGAACCCAAGAA-3’	5’-CCTTGTTGGACACATCCTCAC-3’
*TIMP1*	5’-CTTCTGCAATTCCGACCTCGT-3’	5’-ACGCTGGTATAAGGTGGTCTG-3’
*TIMP2*	5’-GCTGCGAGTGCAAGATCAC-3’	5’-TGGTGCCCGTTGATGTTCTTC-3’
*HPSE*	5’-TCCTGCGTACCTGAGGTTTG-3’	5’-CCATTCCAACCGTAACTTCTCCT-3’
*HAS1*	5’-TCAAGGCGCTCGGAGATTC-3’	5’-CTACCCAGTATCGCAGGCT-3’
*HAS2*	5’-TCCTGGATCTCATTCCTCAGC-3’	5’-TGCACTGAACACACCCAAAATA-3’
*HAS3*	5’-CGCAGCAACTTCCATGAGG-3’	5’-AGTCGCACACCTGGATGTAGT-3’

A list of Primer Sequences Used for PCR is Shown in Table 1.

### Inflammatory mediators

Human Interleukin-6 (BD Biosciences, San Diego, CA), tumor necrosis factor-α (Novus Biologicals, Littleton, CO), and interferon-γ levels (BD Biosciences, San Diego, CA) were measured using enzyme-linked immunosorbent assays per manufacturer recommendations.

### Statistical analysis and power analysis

Glycocalyx staining intensity and RNA levels are presented as means ± SDs. A *p* value of <0.05 was considered significant for all tests. For comparisons of more than two groups, one-way analysis of variance was first performed, and if *p* < 0.05, pairwise comparisons were performed using a one-tailed Student’s *t* test. All figures show means with error bars that demonstrate SD. Based on previous data showing a difference of approximately 6% and SD of 33% of the mean value in injured HUVECs^8^, a power analysis and sample size determination were performed. With a significance level (*α*) of 0.05 and power (1 − *β*) value of 0.8 that was calculated, a sample number of 475 was required to achieve the 0.8 power value. For subsequent experiments using only high dose of virus, the difference was assumed to be 12%, yielding a required sample size of 120.

## Results

### Glycocalyx degradation after H1N1 infection

**Fig 1:**
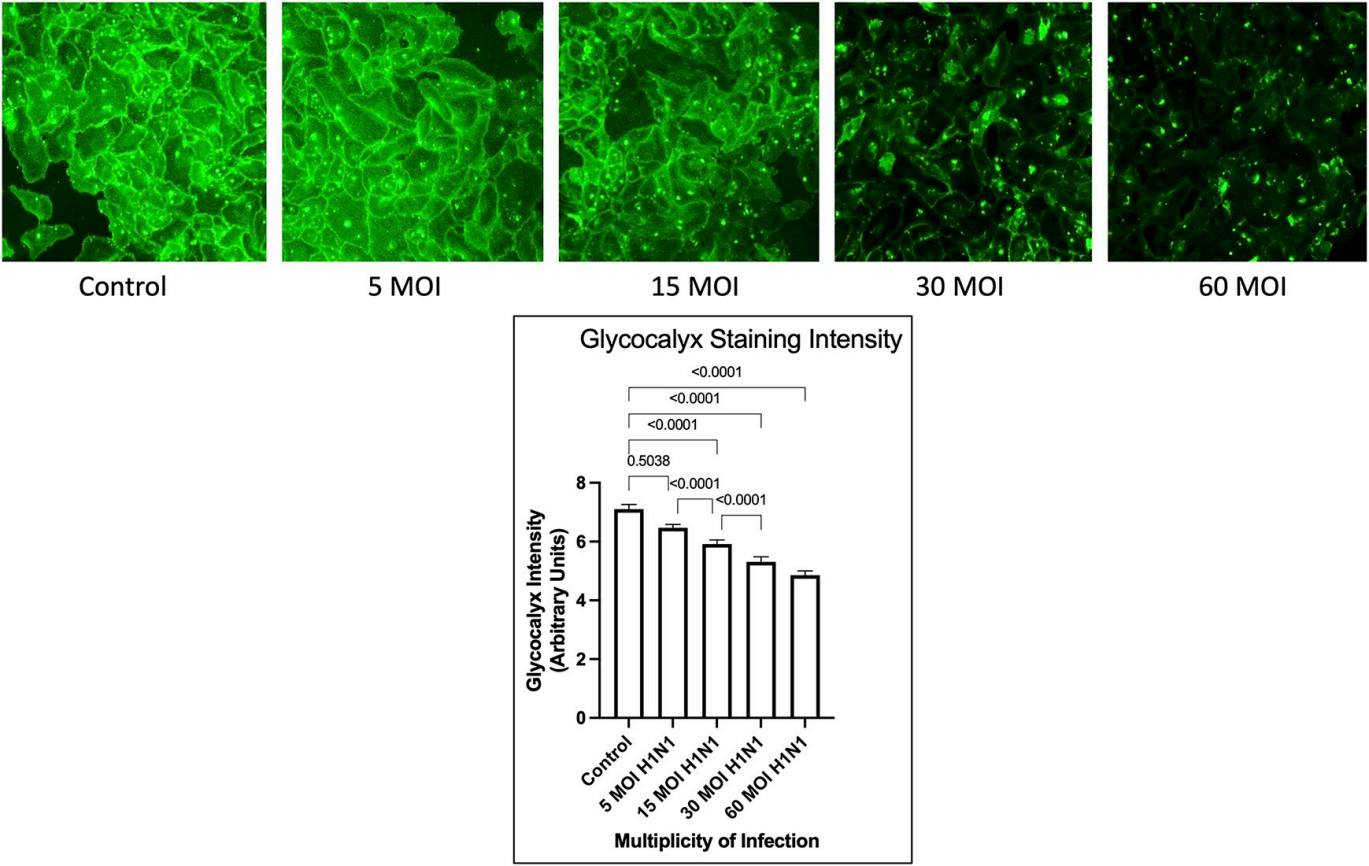
Endothelial glycocalyx staining intensity after H1N1 Infection. A comparison of glycocalyx staining intensity for control and H1N1 influenza virus exposed HUVECs at a multiplicity of infection of 5, 15, 30, and 60 along with representative images.

**Fig 2:**
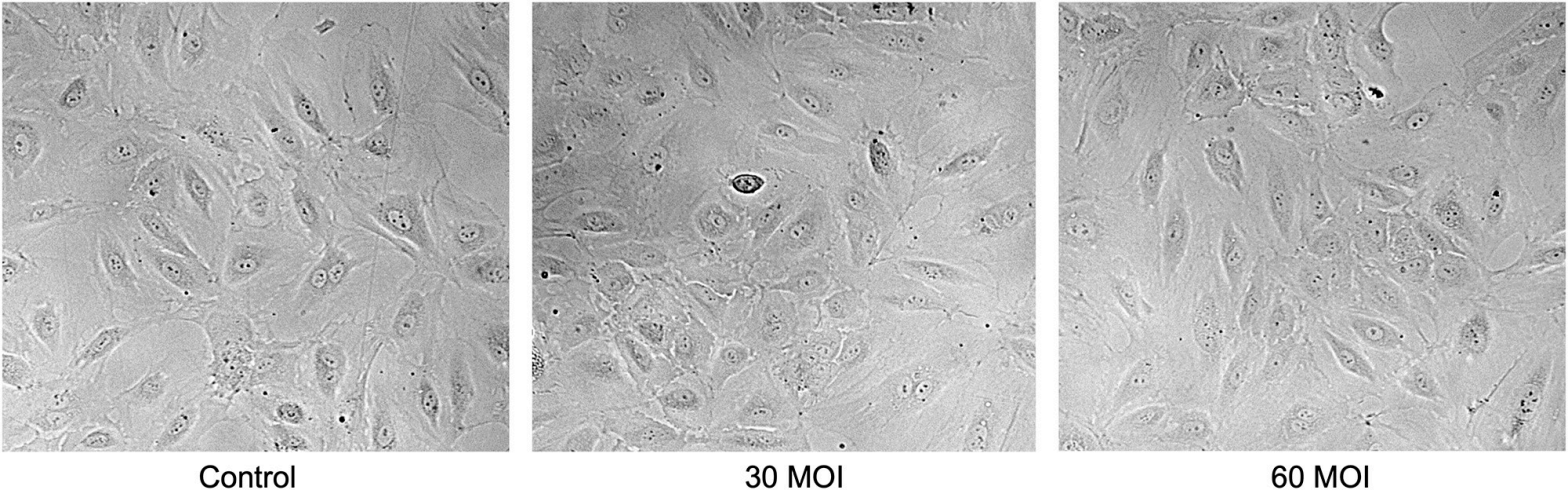
Examining viability of cells. Phase contrast images were taken of the human umbilical vein endothelial cells and showed no overt cytopathology and appeared to be viable.

As shown in Fig 1, the H1N1 virus caused a decrease in endothelial glycocalyx staining intensity in a dose dependent response. This dose dependent response appears to maximize at a multiplicity of infection of 30, as there was no difference in glycocalyx staining intensity when comparing MOI of 30 to 60 (5.31 vs. 4.86 Arbitrary Units, p = 0.13). Phase contrast images were taken to assess overt cytopathology and apoptosis as shown in Fig 2. This demonstrated that the H1N1 infected HUVECs were viable.

**Fig 3:**
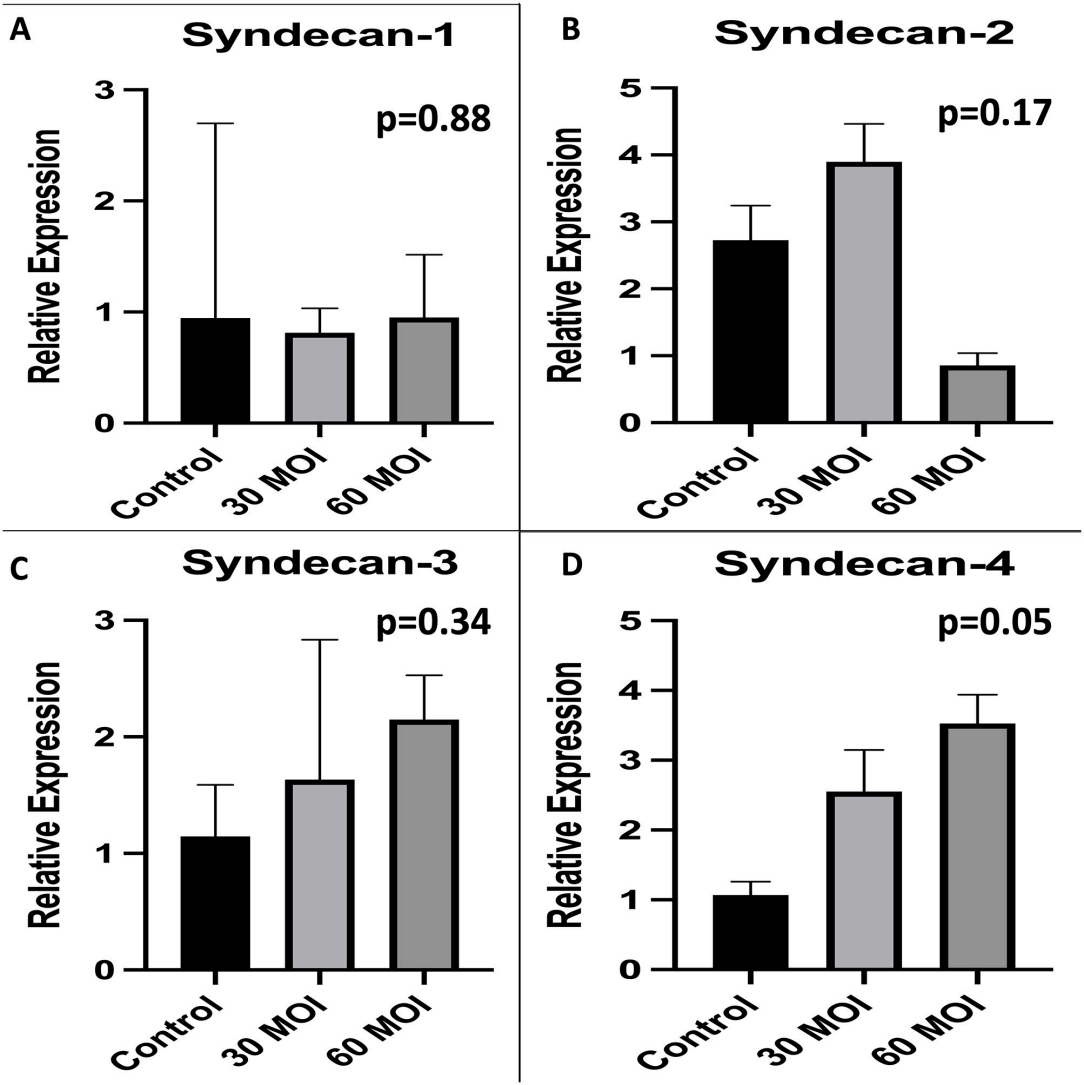
Comparison of syndecan levels. A comparison of syndecan expression as measured by real-time PCR demonstrating that there is no difference in A) syndecan-1, B) syndecan-2, C) syndecan-3, and 4) syndecan-4 expression when H1N1 infected human umbilical vein endothelial cells are compared to control.

To measure markers of glycocalyx degradation, we examined Syndecan levels. As shown in Fig 3, there was no difference in Syndecan-1, -2, -3, and -4 levels after H1N1 infection of HUVECs.

### Matrix metalloproteinases

**Fig 4:**
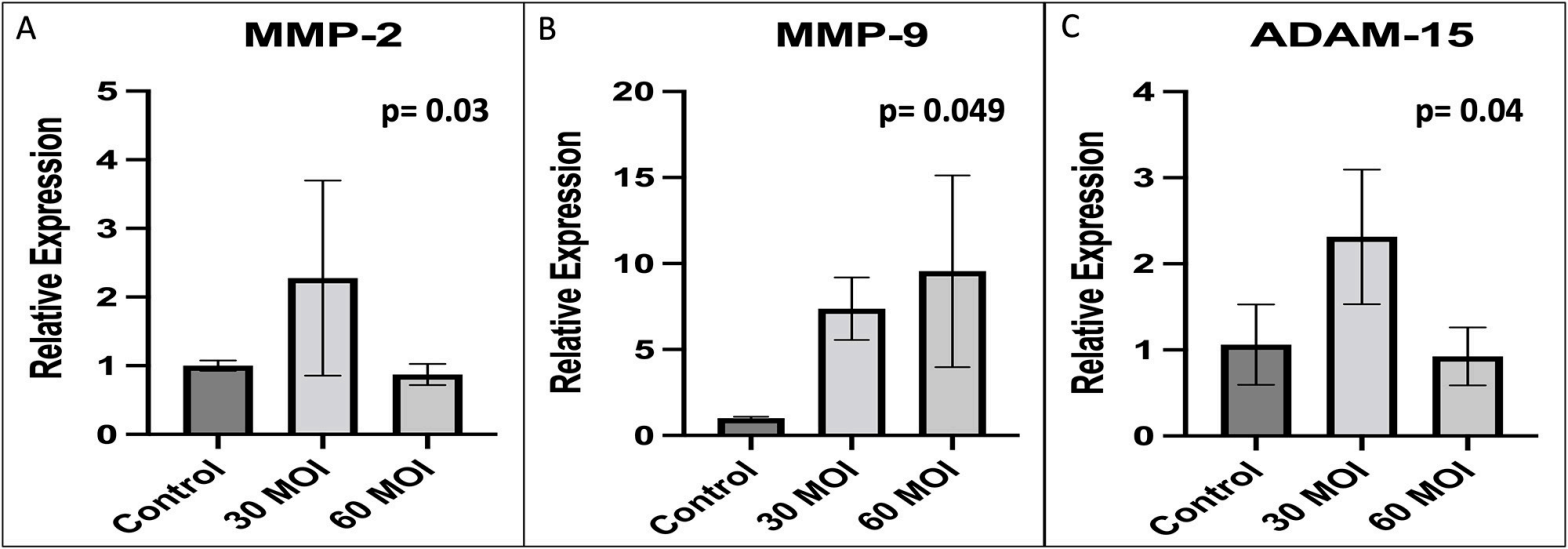
Comparison of metalloproteinase levels. A comparison of metalloproteinase expression as measured by real time PCR demonstrating that A) MMP-2, B) MMP-9, and C) ADAM-15 are increased in HUVECs after H1N1 infection at multiplicity of infection of 30 and 60.

**Fig 5:**
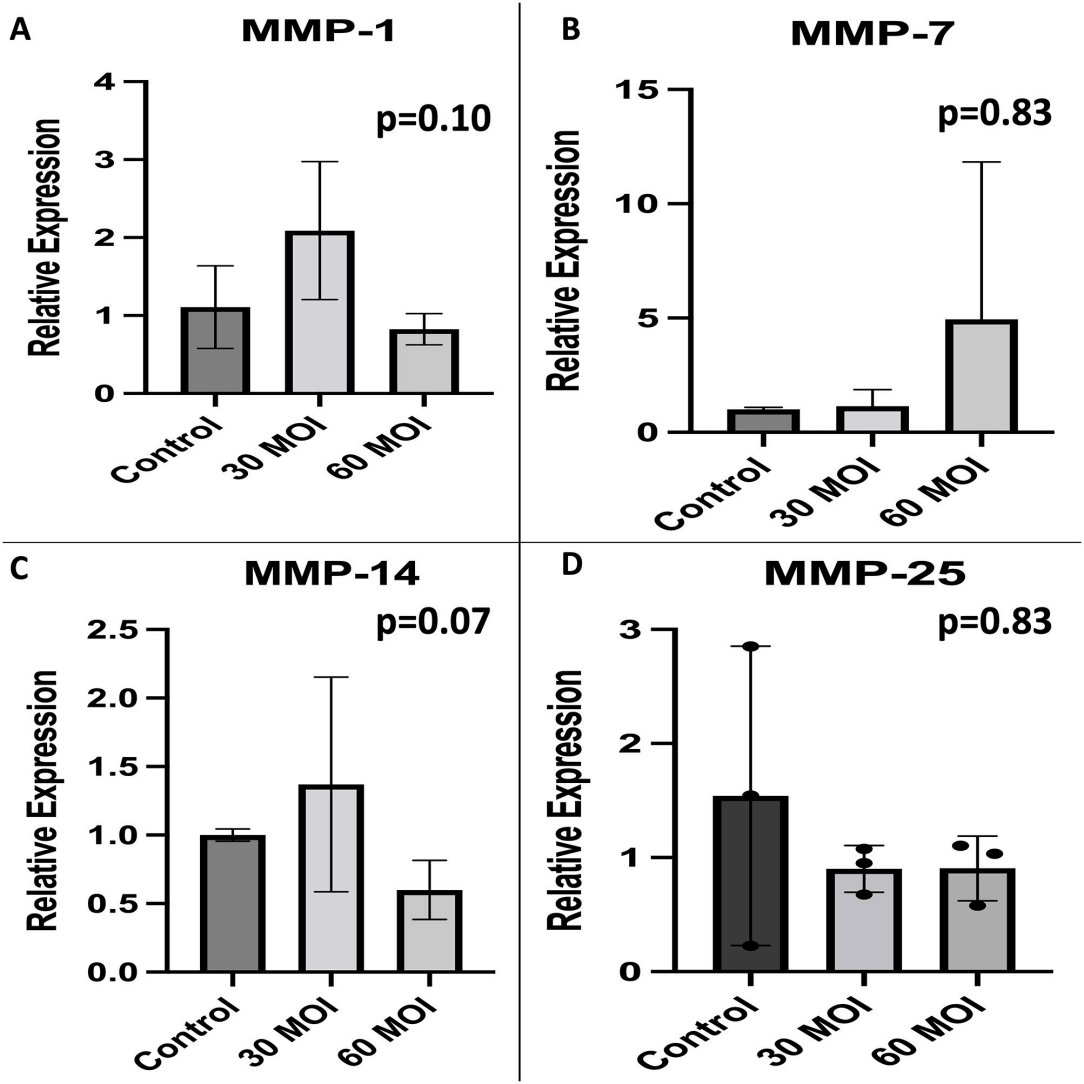
Comparison of metalloproteinase levels. A comparison of metalloproteinase expression as measured by real-time PCR demonstrating that there is no difference in A) MMP-1, B) MMP-7, C) MMP-14, and 4) MMP-25 expression when H1N1 infected human umbilical vein endothelial cells are compared to control.

Because matrix metalloproteinases (MMP) are known to mediate glycocalyx degradation, we examined MMP levels by real-time PCR. As shown in Fig 4A, MMP-2 levels were increased at an MOI of 30. MMP-9 levels appeared to increase in a dose dependent response as shown in Fig 4B. In addition, ADAM-15 levels were increased at an MOI of 30 (Fig 4C). As shown in Fig 5, there was no difference in levels of MMP-1, MMP-7, MMP-14, and MMP-25. ADAM-17 levels were also found to not be different at an MOI of 30 and 60 when compared to control HUVECs (p = 0.23).

**Fig 6:**
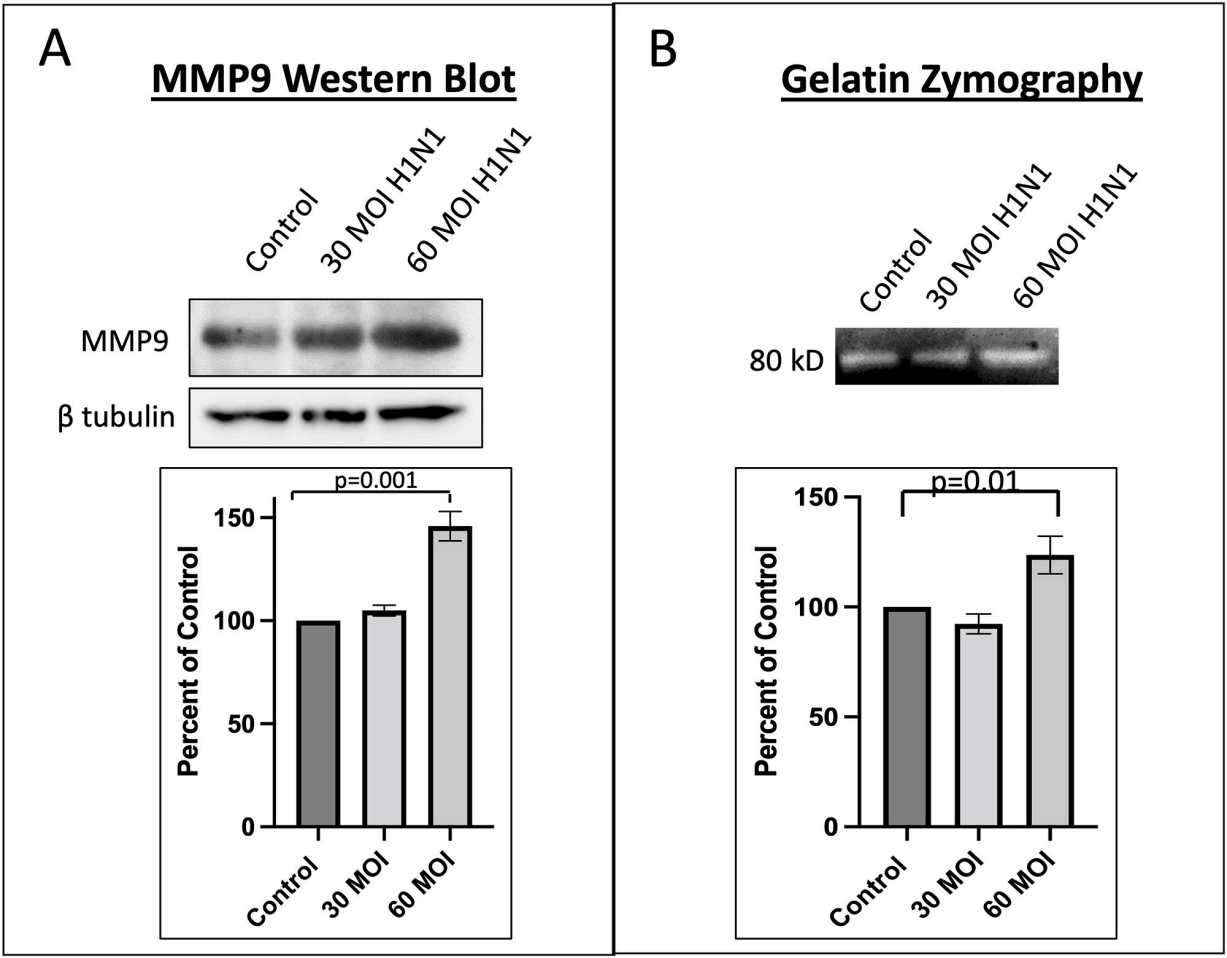
MMP-9 levels as measured by western blot and zymography. A comparison of MMP-9 in human umbilical vein endothelial cells as measured by A) western blot and B) zymography showing that amount of MMP-9 is increased as compared to control when H1N1 infection occurs with multiplicity of infection of 60.

To further examine the role of MMP-9, we performed western blot and gelatin zymography analysis with HUVECs infected with H1N1 virus at an MOI of 30 and 60 as shown in Fig 6. MMP-9 levels were found to be highest at a MOI of 60 by western blot (Fig 6A) and by gelatin zymography (Fig 6B).

**Fig 7:**
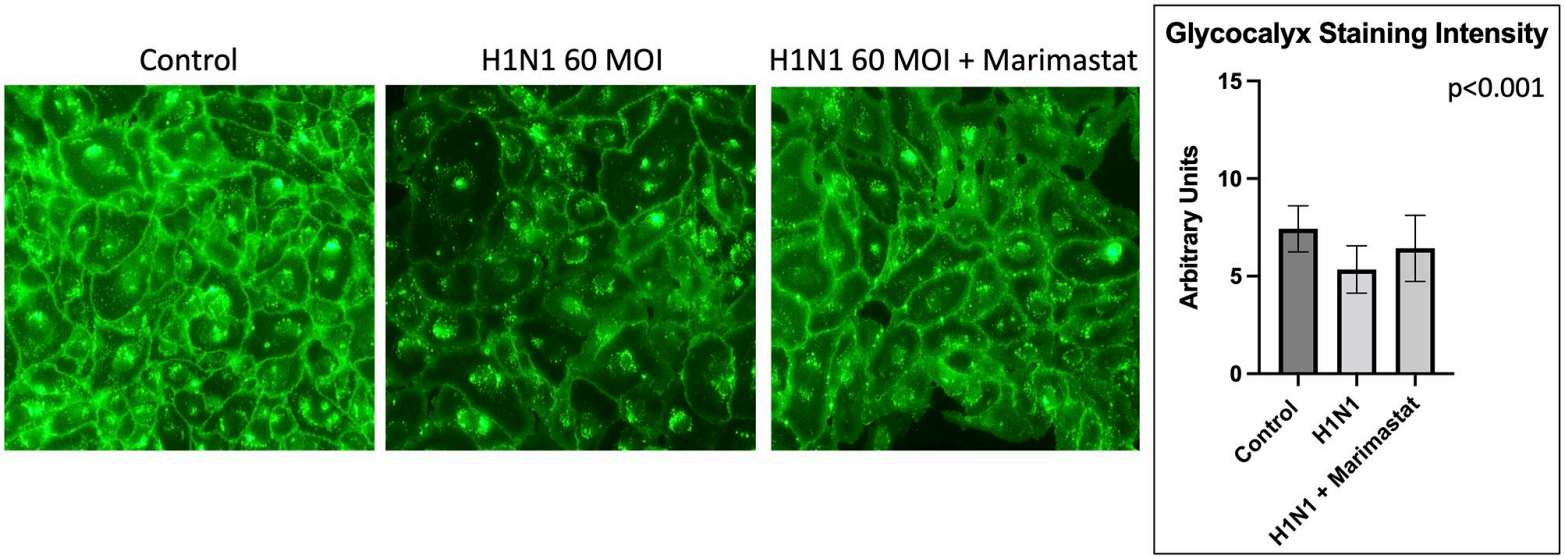
Metalloproteinase inhibitors protect the glycocalyx. Representative images and comparison of glycocalyx staining intensity shows that glycocalyx staining intensity is preserved when H1N1 infection occurs in the presence of the metalloproteinase inhibitor marimastat for human umbilical vein endothelial cells.

To further inspect the role of MMPs in the endothelial glycocalyx degradation seen in H1N1 infection, we infected HUVECs with H1N1 at an MOI of 60 with and without the MMP inhibitor Marimastat. As shown in Fig 7, Marimastat protected the endothelial glycocalyx from degradation when compared to H1N1 infection alone (6.43 vs. 5.34, p<0.001).

### Pro-glycocalyx agents

**Fig 8:**
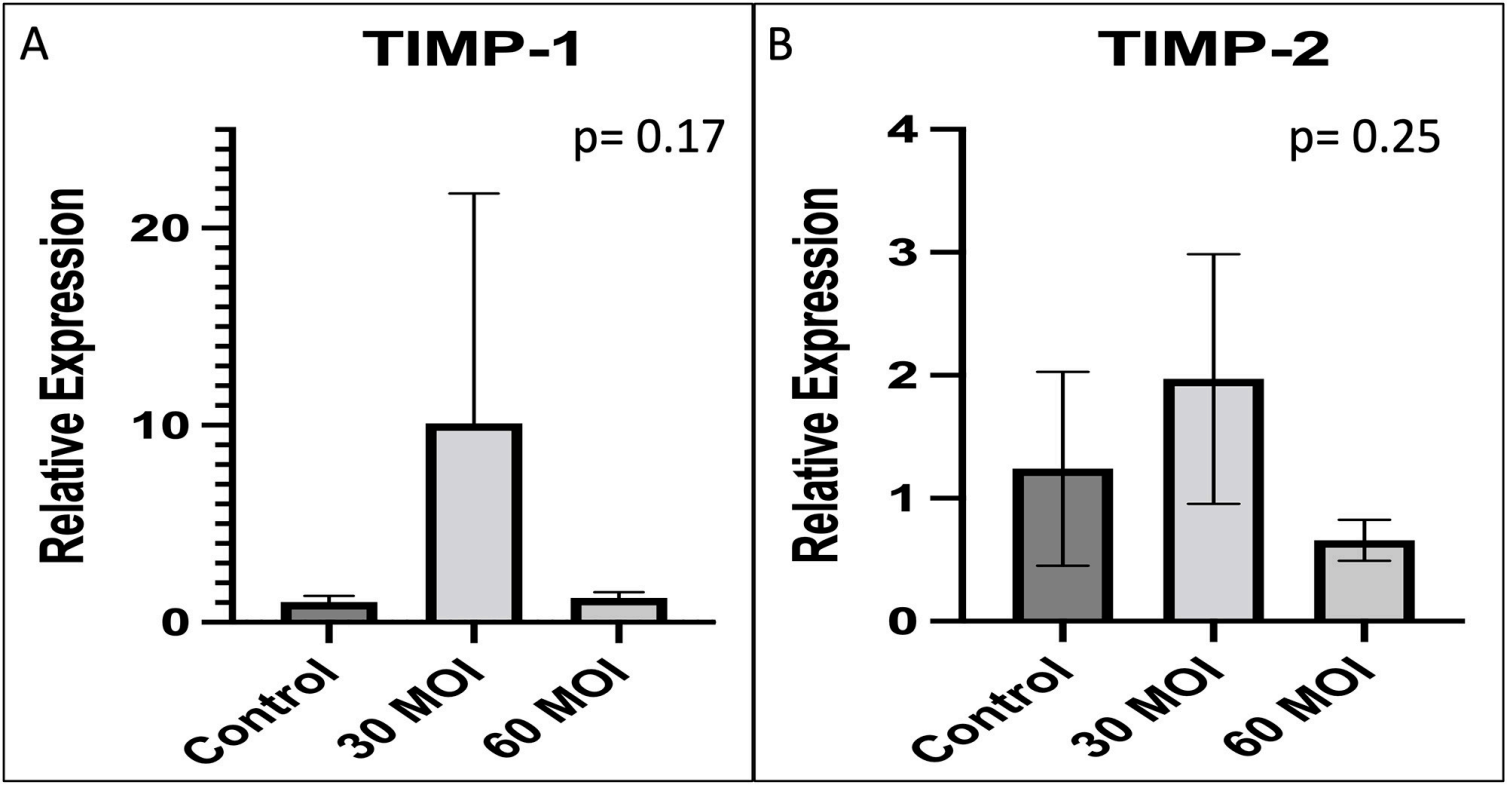
Comparison of pro-glycocalyx agents. A comparison of pro-glycocalyx agent expression as measured by real-time PCR demonstrating that there is no difference in A) TIMP-1 and B) TIMP-2 when H1N1 infected human umbilical vein endothelial cells are compared to control.

**Fig 9:**
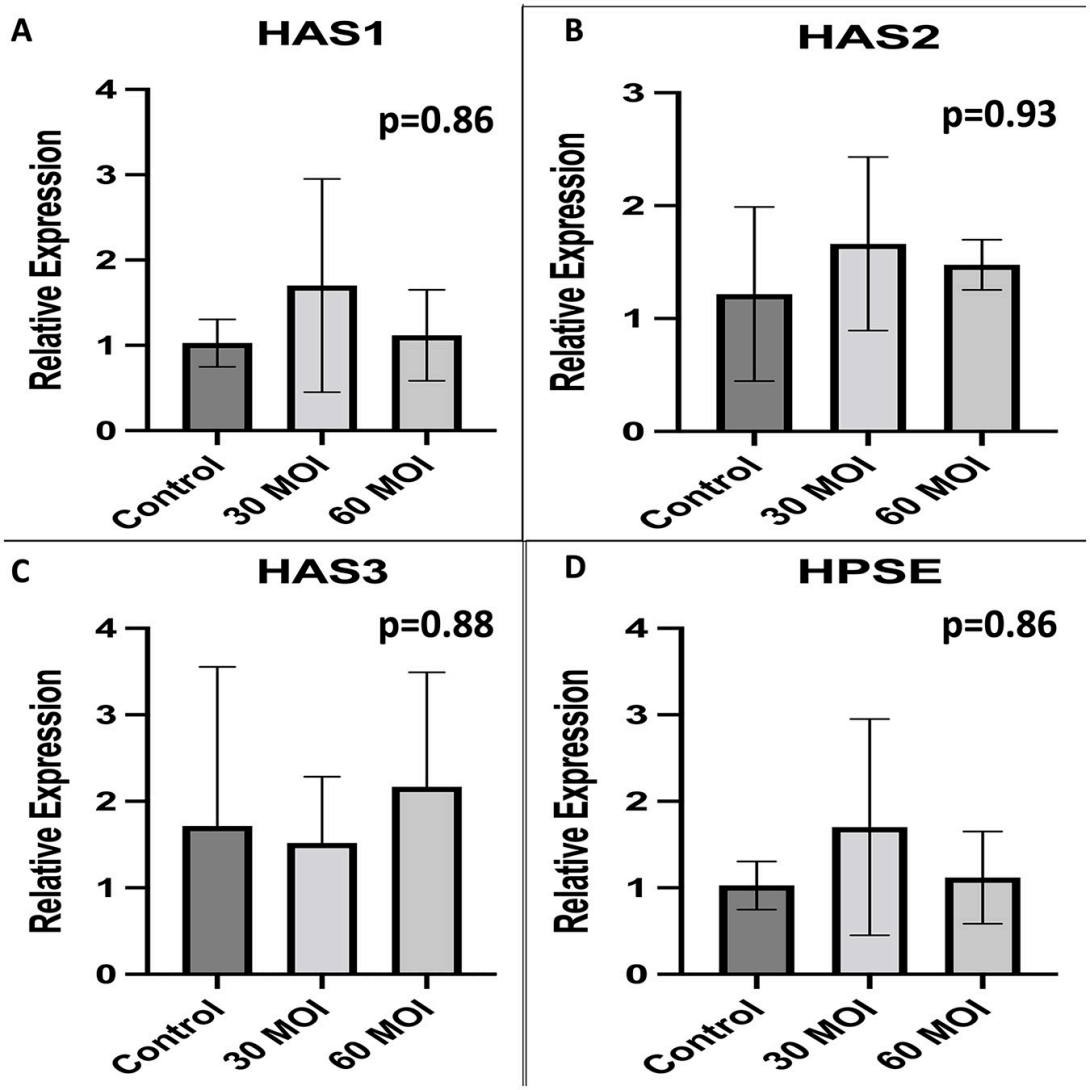
Pro-glycocalyx agents after H1N1. A comparison of pro-glycocalyx agent expression as measured by real-time PCR demonstrating that there is no difference in A) HAS1 and B) HAS2 or C) HAS3 when H1N1 infected human umbilical vein endothelial cells are compared to control. In addition, there is no difference in heparinase expression as measured by real-time PCR.

Tissue Inhibitor of Metalloproteinases-1 (TIMP-1) and TIMP-2 were measured by PCR and found to be no different as shown in Fig 8. Hyaluronic Acid Synthase-1 (HAS-1), HAS-2, and HAS-3 expression were found to be no different with H1N1 infection as shown in Fig 9A–9C. Heparanase expression was also found to be no different with H1N1 infection in HUVECs as seen in Fig 9D.

### Role of neuraminidase

**Fig 10:**
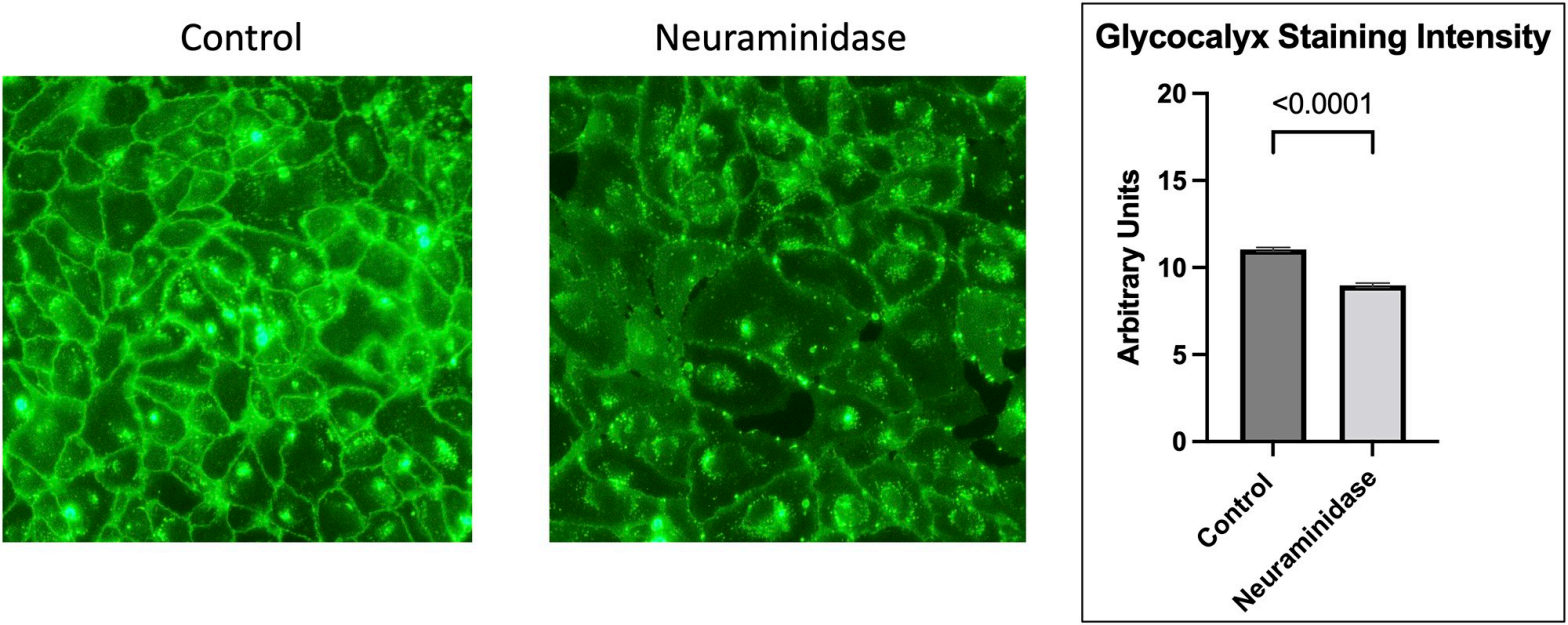
Neuraminadase’s role in glycocalyx shedding. Representative images and comparison of glycocalyx staining intensity shows that neuraminidase alone causes decreased glycocalyx staining intensity when compared to control human umbilical vein endothelial cells.

**Fig 11:**
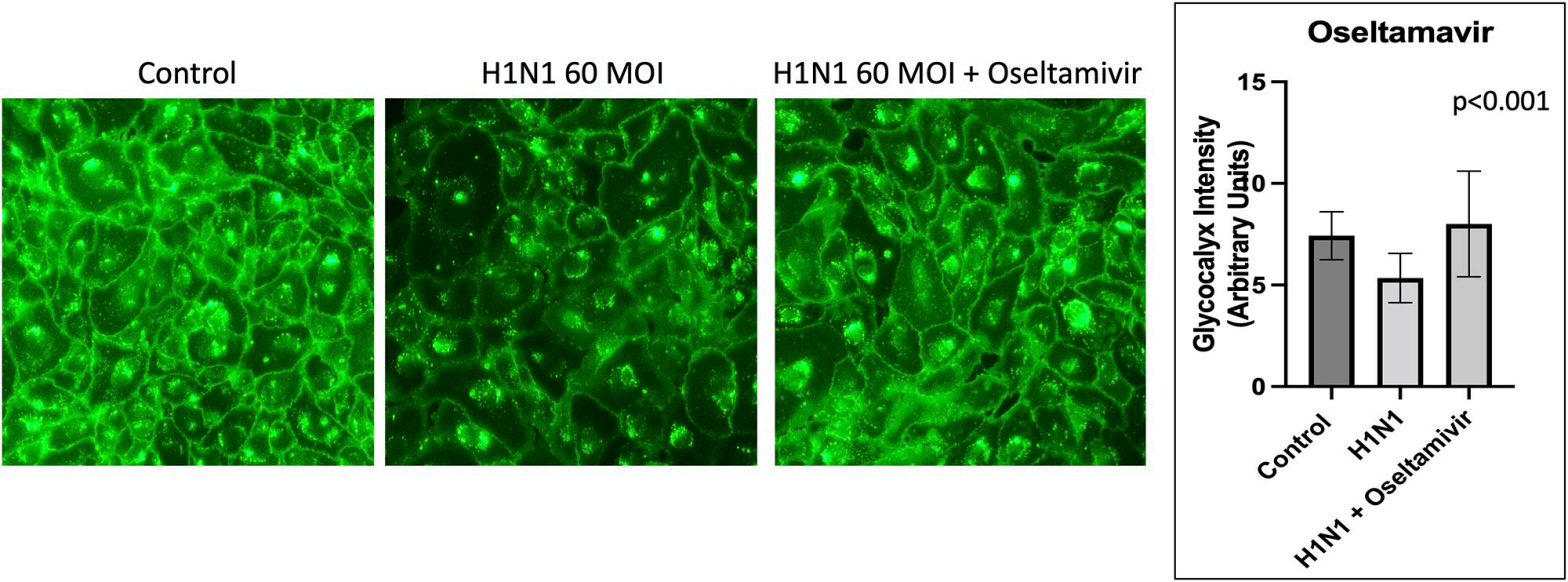
Neuraminidase inhibitors protect the glycocalyx. Representative images and comparison of glycocalyx staining intensity in human umbilical vein endothelial cells shows that glycocalyx staining intensity is preserved when neuraminidase exposure occurs in the presence of the neuraminidase inhibitor Osteltamavir.

Neuraminidase is a component of the H1N1 influenza virus and can cleave sialic acid, an important component of the endothelial glycocalyx. To examine the role of neuraminidase, we exposed HUVECs to recombinant neuraminidase. As shown in Fig 10, neuraminidase alone results in decreased endothelial glycocalyx staining intensity when compared to control (11.04 vs. 8.98 Arbitrary Units, p<0.001). To further characterize the role of neuraminidase in viral glycocalyx degradation, we exposed HUVECs to a neuraminidase inhibitor, Oseltamivir, concomitantly with H1N1 viral infection. As shown in Fig 11, Osteltamavir resulted in preserved endothelial glycocalyx staining after H1N1 infection as compared to H1N1 infection alone (5.34 vs. 8.00 Arbitrary Units, p<0.001).

### Inflammatory mediators

**Fig 12:**
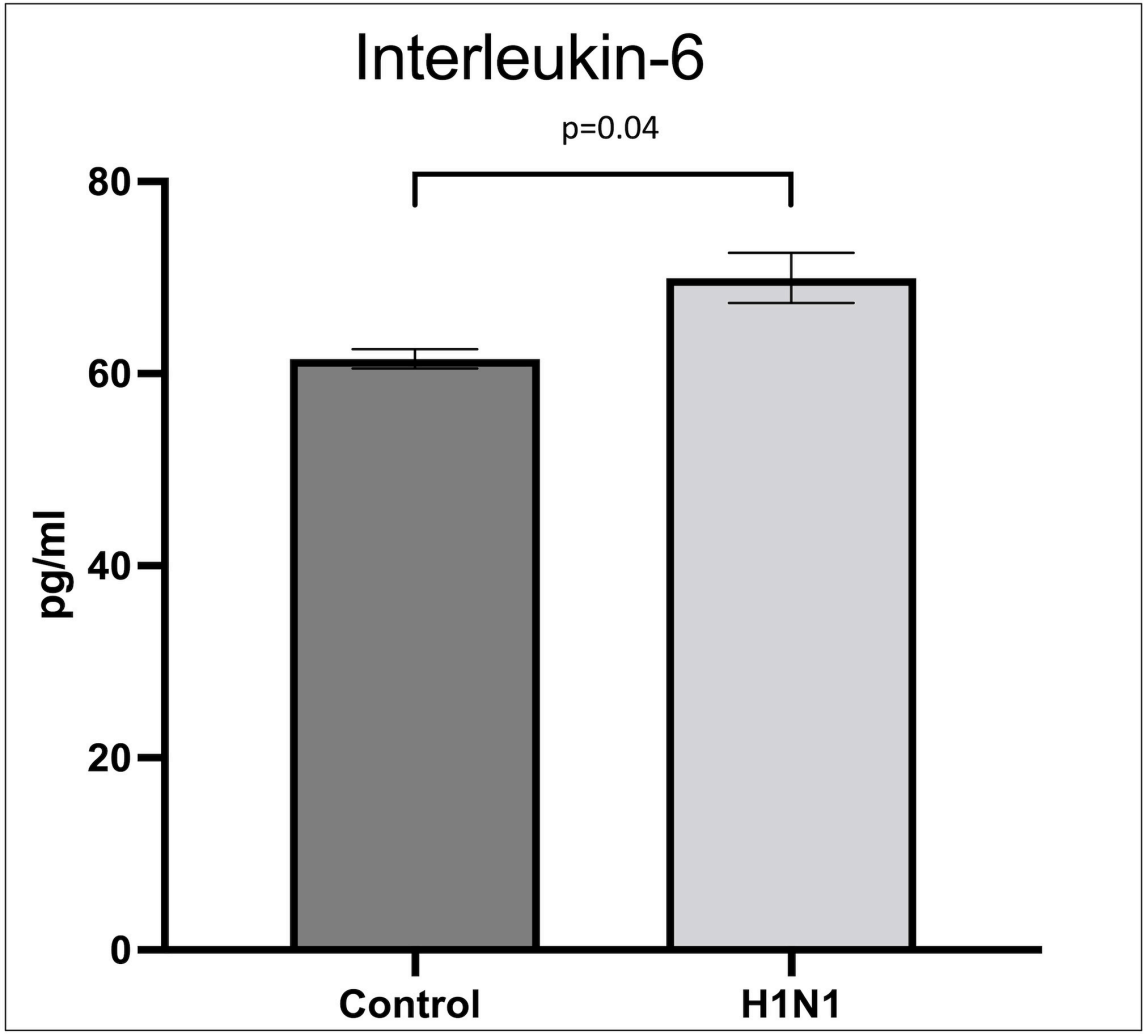
Comparison of Interleukin-6 levels. Interleukin-6 levels are increased in HUVECs after H1N1 influenza infection when compared to control.

Interleukin-6 (IL-6) levels were higher in HUVECs exposed to H1N1 as shown in Fig 12 when compared to control. Tumor necrosis factor-α and interferon-γ levels were not different in H1N1 exposed HUVECs and were found in undetectable quantities in both the control and H1N1 exposed groups.

## Discussion

Viral endotheliopathy has taken on increased interest in the current COVID-19 pandemic. How the H1N1 influenza virus affects the endothelial glycocalyx is poorly understood. In this study, we examined how the H1N1 virus induces decreased endothelial glycocalyx staining intensity and the mechanism by which it increases glycocalyx shedding.

We found that the H1N1 virus causes a decrease in glycocalyx staining intensity. Prior work has established this link, as clinical studies have shown that markers of endothelial glycocalyx degradation correlate with poor prognosis after H1N1 infection [17,18]. However, because these studies were clinical, the endothelial glycocalyx layer itself could not be imaged. Furthermore, it was unclear whether H1N1 could damage the glycocalyx directly, or whether this correlation was due to general immune system activation or other indirect effects. Prior work has shown that it is the *epithelial* glycocalyx that is first affected by viral infection. And that it is mainly the degradation products of the epithelial glycocalyx and consequent epithelial permeability that leads to destruction of the endothelial glycocalyx [25]. Because of the nature of our study using HUVECs, we could not examine the role of the epithelial cells in H1N1 infection. While prior work has shown decreased glycocalyx staining intensity after bacterial infection [26], ours is the first to directly show that H1N1 infection results in decreased staining intensity of the endothelial glycocalyx. In addition, we found that this occurs in a dose dependent manner, with higher MOIs resulting in more severe decrease in glycocalyx staining intensity. This dose-dependent response is consistent with other studies that have shown worse effects with higher MOI of H1N1 infection. For example, animal studies have shown that increased multiplicity of infection for H1N1 and H3N2 influenza virus results in increased levels of cell death and apoptosis [27]. How endothelial glycocalyx degradation contributes to pneumonia and ARDS after H1N1 infection needs further investigation.

MMPs are known to be an important mediator of the endothelial glycocalyx [7,28,29]. We found that MMP-2, MMP-9, and ADAM-15 may play a key role in degradation of the glycocalyx after H1N1 infection. The importance of MMP-2 in regulating the endothelial glycocalyx has been demonstrated in prior studies [21,30]. ADAM-15 plays a key role in glycocalyx degradation after LPS injury [31]. In our study, we found that MMP-9 in particular, plays an important role in the dose dependent response of endothelial glycocalyx degradation in H1N1 infection. MMP-9 plays a key role in other viral induced glycocalyx degradation, such as in dengue fever [32]. MMP-9 has also been found in increasing serum and lung levels after influenza A infection [33]. MMP-9 also causes endothelial glycocalyx degradation after LPS injury [21]. Novel therapies targeting MMPs and particularly MMP-9 may be effective against H1N1 infection.

The present study showed that neuraminidase plays a role in degrading the endothelial glycocalyx. Neuraminidase cleaves sialic acid, an important component of the glycocalyx layer [34]. Neuraminidase is known to be a potent enzyme for removing sugar residues from the glycocalyx of glomerular endothelial cells [35]. Our work is consistent with prior studies that have shown neuraminidase can degrade the glycocalyx in HUVECs [36]. Anti-virals like Oseltamivir work through inhibition of neuraminidase. In this study, we found that Oseltamivir protected the endothelial glycocalyx in the face of H1N1 infection. Further studies are needed to determine whether neuraminidase inhibitors may protect the glycocalyx in other disease states.

Inflammatory mediators are also known to cause glycocalyx degradation in viral infection. Plasma taken from patients infected with COVID-19 was found to be higher in interleukin-6 (IL-6). This plasma induced glycocalyx shedding in HUVECs, while addition of low molecular weight heparin inhibited glycocalyx damage [37]. We found that H1N1 infection in HUVECs also increased IL-6 levels and increased IL-6 levels may have contributed to the glycocalyx damage seen in the present study. Sulfation also plays a key role in maintenance of the endothelial glycocalyx. Under sulfated glycocalyx may increase susceptibility to COVID-19 infection and result in worsening shedding of the glycocalyx [25]. For this reason, we used media (M200 media) that contains inorganic sulfate, which is the limiting factor to sulfation of the glycocalyx.

Maintenance of the endothelial glycocalyx layer is a dynamic process, with shedding and synthesis of the glycoprotein layer occurring concomitantly. Importantly, this study demonstrated that H1N1 infection does not appear to impact the synthesis process but does increase degradation. Hyaluronic acid synthase (HAS) genes expression did not change with H1N1 infection. HAS is a pro-glycocalyx agent that helps synthesis the glycoprotein layer. Increasing glycocalyx synthesis in the face of viral illness may represent another potential therapy to mitigate glycocalyx damage. Various studies have examined methods to protect the glycocalyx by increasing HAS levels [38,39].

This study was not without limitations. This study was done using HUVECs in a cell culture model. Therefore, several physiologic and pathologic stimuli could not be replicated in our model. Prior studies have shown that in vivo studies of the endothelial glycocalyx differ from physiological findings [9]. Studying the structural and functional properties of the endothelial glycocalyx using static imaging can represent a narrow possibility and newer multi-height microfluidic platforms have been developed [40]. However, our previous work has shown flow conditions do not change regulation of glycocalyx shedding [3]. In vivo studies are needed to confirm our findings. In addition, we used HUVECs because they are commonly used to study endothelial cell pathways. However, other endothelial cell types, such as in lung capillaries, may demonstrate different responses in viral infection.

## Conclusions

In conclusion, we found that the H1N1 infection results in degradation of the endothelial glycocalyx using various MMPs. MMP-9 appears to play a particularly important role in this process. Furthermore, neuraminidase also contributes to the shedding of endothelial glycocalyx. Further studies are needed to define the role of the endothelial glycocalyx damage in significant lung injury after acute viral infection.

## Supporting information

S1 Raw images (PDF)
