# Supplementary figures and images for: Glycocalyx degradation and the endotheliopathy of viral infection
Source: PLoS One. 2022 Oct 19;17(10):e0276232. doi: 10.1371/journal.pone.0276232 (PMC9581367; doi:10.1371/journal.pone.0276232)

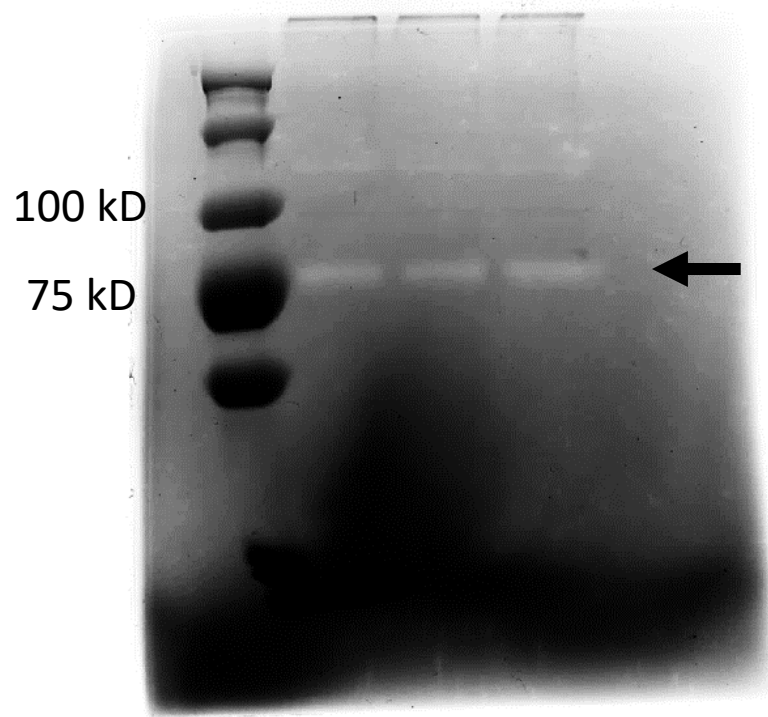

Gelatin zymography

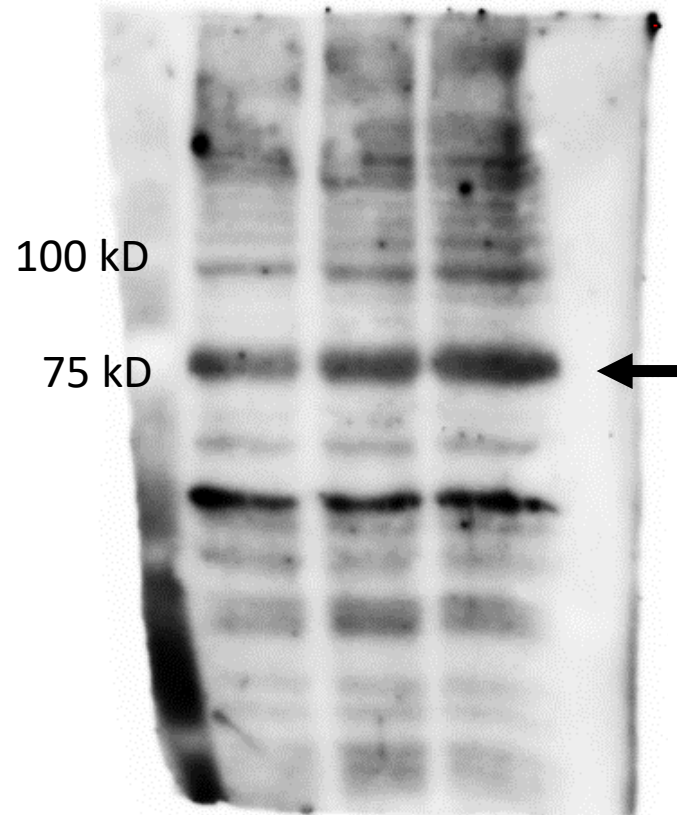

MMP9 western blot

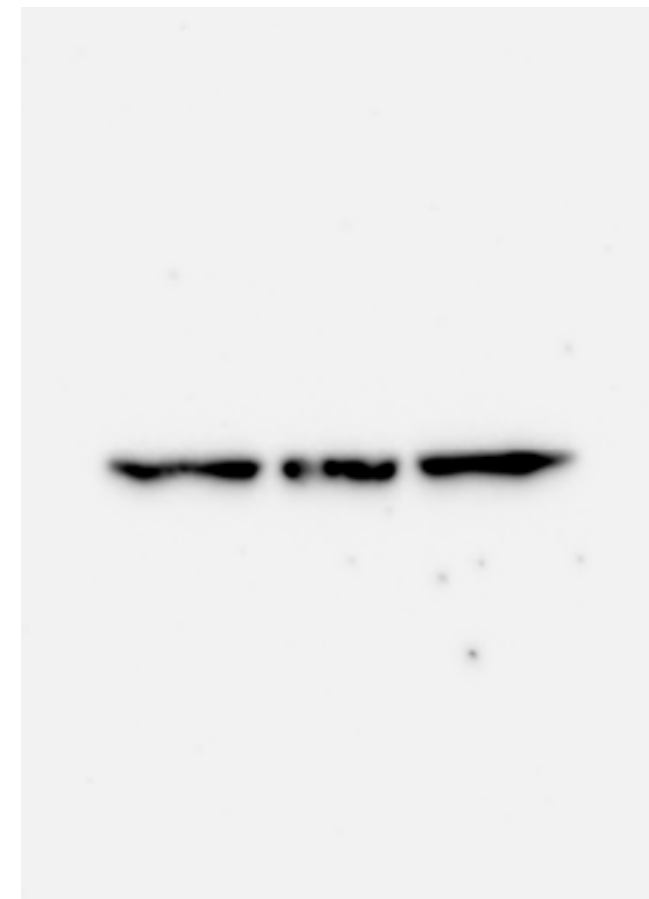

$\beta$  Tubulin western blot

Supplement: S1 Raw images — (PDF) [file pone.0276232.s001.pdf]
